# Phenotypic analysis of the Arabidopsis heat stress response during germination and early seedling development

**DOI:** 10.1186/1746-4811-10-7

**Published:** 2014-03-10

**Authors:** Joana Silva-Correia, Sara Freitas, Rui M Tavares, Teresa Lino-Neto, Herlânder Azevedo

**Affiliations:** 1 Center for Biodiversity, Functional & Integrative Genomics (BioFIG), Plant Functional Biology Center, University of Minho, Campus de Gualtar, 4710-057 Braga, Portugal; 2 Present address: 3B’s Research Group – Biomaterials, Biodegradables and Biomimetics, University of Minho Headquarters of the European Institute of Excellence on Tissue Engineering and Regenerative Medicine, AvePark, 4806-909 Guimarães, Taipas, Portugal; 3 Present address: CIBIO, Centro de Investigação em Biodiversidade e Recursos Genéticos, Universidade do Porto, Campus Agrário de Vairão, 4485-661 Vairão, Portugal

**Keywords:** Arabidopsis, Heat stress, Mutants, Phenotype characterization, Thermotolerance

## Abstract

**Background:**

Phenotypic characterization of *Arabidopsis thaliana* gain- and loss-of-function mutants is a delicate and meticulous task that often involves the analysis of multiple parameters. Arabidopsis heat tolerance has been evaluated based on direct assessments that include seed germination, seedling survival, hypocotyl and root elongation, or indirect measurements such as chlorophyll content or ion leakage.

**Results:**

In an attempt to simplify the detection of heat stress-associated phenotypes, a collection of protocols for analysis of seed germination and seedling survival to heat treatment is proposed. Temperatures and lengths of heat treatments were combined into several heat tolerance assays, to be used as a primary approach for the search and characterization of basal and acquired heat tolerance-associated phenotypes at early developmental stages. The usefulness of this methodology was illustrated through the characterization of heat-related phenotypes in different Arabidopsis ecotypes as well as in gain- and loss-of-function mutants.

**Conclusions:**

The use of standardized experimental protocols designed to detect temperature-related phenotypes is proposed. The suggested plate-based assays provide an appropriate framework of experimental conditions for detection of variability amongst natural accessions or mutants lines. Functional studies could be facilitated by using this inexpensive and undemanding approach.

## Background

With the onset of post-genomic capabilities in *Arabidopsis thaliana*, and particularly the availability of large collections of genome-indexed insertion mutants, the plant biology community has been thoroughly employing reverse genetic strategies [1]. The most critical step in this approach is the identification of a phenotype that is distinct from the wild-type, thus providing new insights on gene function [2,3]. Searching for a noticeable phenotype can be a strenuous process, often hindered by gene redundancy, natural variability, or subtle imperceptible changes in growth or development [4]. Previously, Boyes et al. [5] developed a sensitive and dynamic method to detect and interpret phenotypic differences over the entire span of Arabidopsis development, based on a time-dependent map of well-defined morphological traits. However, detection of differential phenotypes is often dependent on the application of particular experimental conditions, specific environmental challenges or a combination of several inputs [4]. Thus, selection of the most suitable experimental conditions is critical for success in the identification of a phenotype.

In the specific case of heat tolerance, a standardized experimental protocol designed to detect temperature-related phenotypes has not been reported to the best of our knowledge. Distinct assays have been used by different laboratories to distinguish responses to temperature, including the analysis of seedling survival [6-14], adult plant survival/fresh weight [9,15], seed germination and cotyledon greening [9,10,13], chlorophyll accumulation [16,17], shoot elongation [15], hypocotyl elongation [6,7,9,18-20], root growth [9,13,17], ion leakage [6], TBARS accumulation [9], and reporter enzyme activity [18]. In addition to the way the phenotype is scored, other variables lead to differentiated thermotolerance assays, including the plant species, the plant’s developmental stage and the type of heat stress regime (reviewed by [21]). The importance of different time and temperature treatments was precisely depicted in pioneer studies in soybean, leading to important advances in the study of heat stress resistance (e.g. [22]). Unfortunately, the use of such different conditions promotes high variability in the results, with implications in the capacity to detect differential phenotypes.

Plants can rapidly acquire heat tolerance to otherwise lethal temperatures, if they are pre-exposed to a moderate high-temperature or exposed to a gradual increase in temperature. This phenomenon, known as acquired heat tolerance, is clearly distinct from basal heat tolerance, which refers to the innate ability of plants to survive exposure to temperatures above those optimal for growth [23]. As differences have also been detected when using different recovery periods between the acclimation treatment and the high temperature challenge, the term ‘thermotolerance diversity’ was proposed to describe the multiple mechanisms that plants use in response to changes in the environment [21]. The type of heat tolerance involved (basal or acquired) and the type of shifting temperature (instant or gradual) must then be considered for the experimental design, in order to enclose most of the potential conditions that could generate a visible phenotype. From the diverse methods used for evaluating thermotolerance phenotypes, the basal thermotolerance and short-term acquired thermotolerance (usually less than 2 h) are the most common forms of phenotyping strategies [21]. Using in vitro assays, the acclimation treatment is usually performed at 37-38°C for 60–90 min [6-9,13,14,17-20]. Temperature and duration of the stress imposition, application of a pre-conditioning treatment (which can differ in temperature and/or in time length) and duration of the recovery period are only some of the conditions that could be arranged in multiple ways to establish a phenotypic assay. An often underestimated aspect is the time point for determining plant traits. Even for similar morphological assessments, some researchers obtain their results a few days after heat treatment, while others wait for several days. Therefore, it is significantly important to perform a time-course analysis of the phenotypic traits in question, which can reveal a phenotype per se, to facilitate the discovery of masked alterations between mutant and wild-type plants. The growth stage of the treated plant also interferes with the recognition of phenotypic alterations. While the analysis performed in seeds is normally restricted to the germination stage, different assessments could be achieved both in early (2.5-10 day-old) or late (10–25 day-old) seedlings.

The diversity of existing protocols compels the creation of comprehensive surveys for heat-associated phenotypes; however these can be time-consuming and unproductive. In order to create a simplified framework for the phenotypic detection of heat-associated mutants, it is necessary to first assess the heat tolerance response of Arabidopsis wild-type plants. Motivated by the same purpose, Burke et al. [16] characterized the acquired heat tolerance of Col seedlings by chlorophyll accumulation assays. However, to our knowledge there is no study that characterizes the heat response of Arabidopsis plants, based on the assessment of important and highly practical germination and seedling survival traits. The present report provides a comprehensive primary approach to identify and interpret phenotypic differences of heat-associated mutants at early developmental stages. In seedlings, basal tolerance was characterized by temperature and exposure time variations, and acquired heat tolerance by characterizing survival after different acclimation conditions. We introduced a seed-based assay that was thoroughly surveyed in terms of temperature and exposure time variations, and subsequently validated by characterizing several natural occurring ecotypes and heat stress-associated functional mutants.

## Results

### Evaluation of basal heat tolerance by seedling survival assays

**Figure 1:**
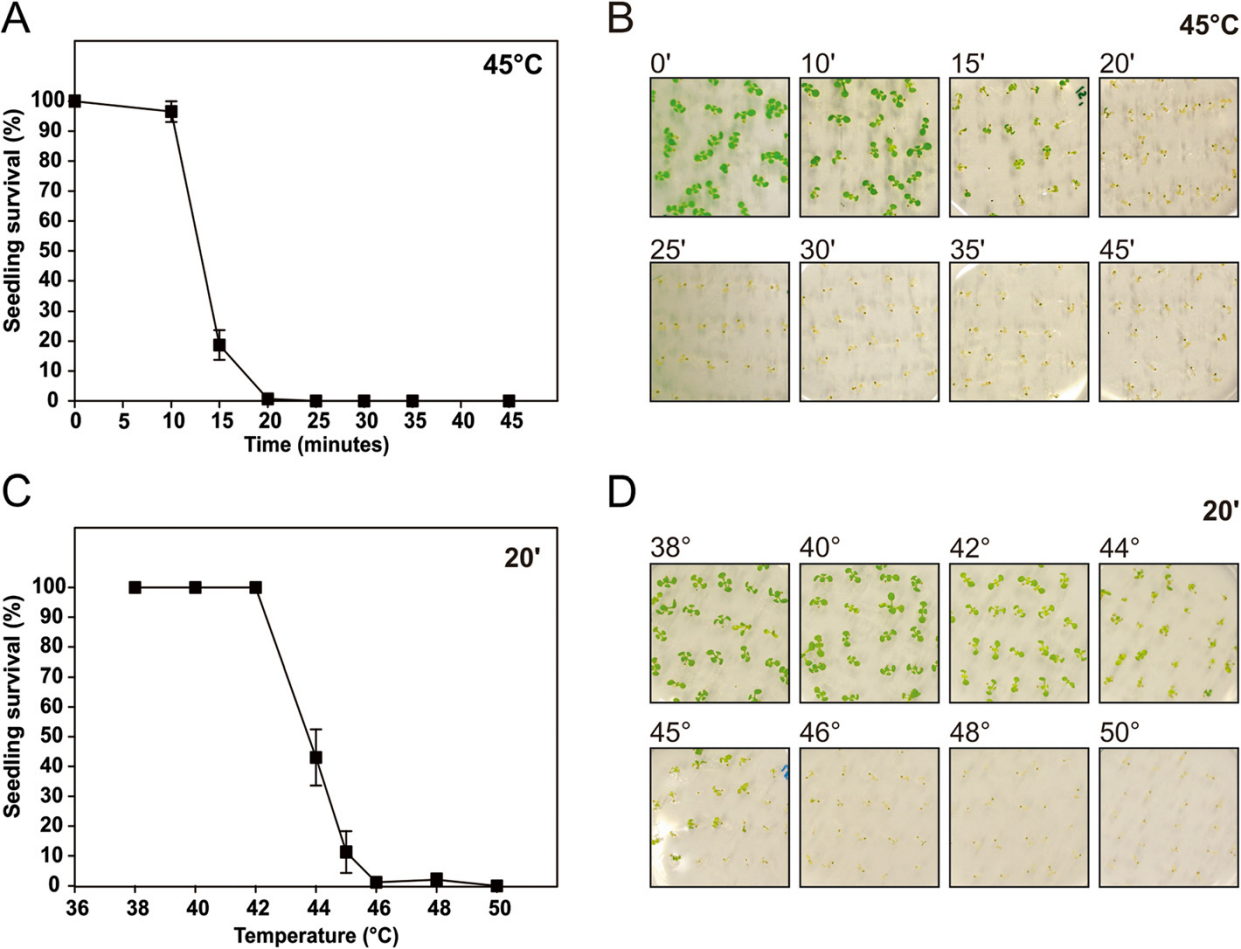
**Assessment of basal heat tolerance in 7-day-old Col seedlings. (A-B)** Seedlings were subjected to a 45°C heat shock for different time periods (0–45 min). **(C-D)** Seedlings were subjected to a 20 min heat shock in a range of different temperatures (38-50°C). Seedling survival was scored after a 6-day recovery period, and represented as a percentage of initial viable seedlings. Error bars represent SEM (n = 5 plates with ~25 seedlings each).

The heat tolerance in photosynthetically active seedlings can be evaluated as a function of seedling survival after imposition of heat stress. Seedlings that remain green and actively growing are considered to be viable. Initial experiments were conducted in the wild-type ecotype Columbia (Col), the reference genotype for Arabidopsis since the sequencing of its genome [24]. Basal heat tolerance in 7-day-old Col seedlings was evaluated in terms of heat shock (HS) duration (10–45 min, at 45°C) and temperature (38-50°C, for 20 min), estimated as the percentage of surviving seedlings after a recovery period of 6 days at 23°C (Figure 1). In order to avoid the introduction of another stress factor, all the assays were performed under a dim light. Results show that, when subjected to HS, seedling viability was lost in a very short time frame (Figure 1A,B): loss was noticeable within 10 min and by min 25 plants became unviable. A similar sigmoidal-like inhibition profile was obtained using 20 min shocks at different HS temperatures (Figure 1C,D). Specifically, seedling viability was lost within the 42-46°C range.

### Evaluation of acquired heat tolerance by seedling survival assays

**Figure 2:**
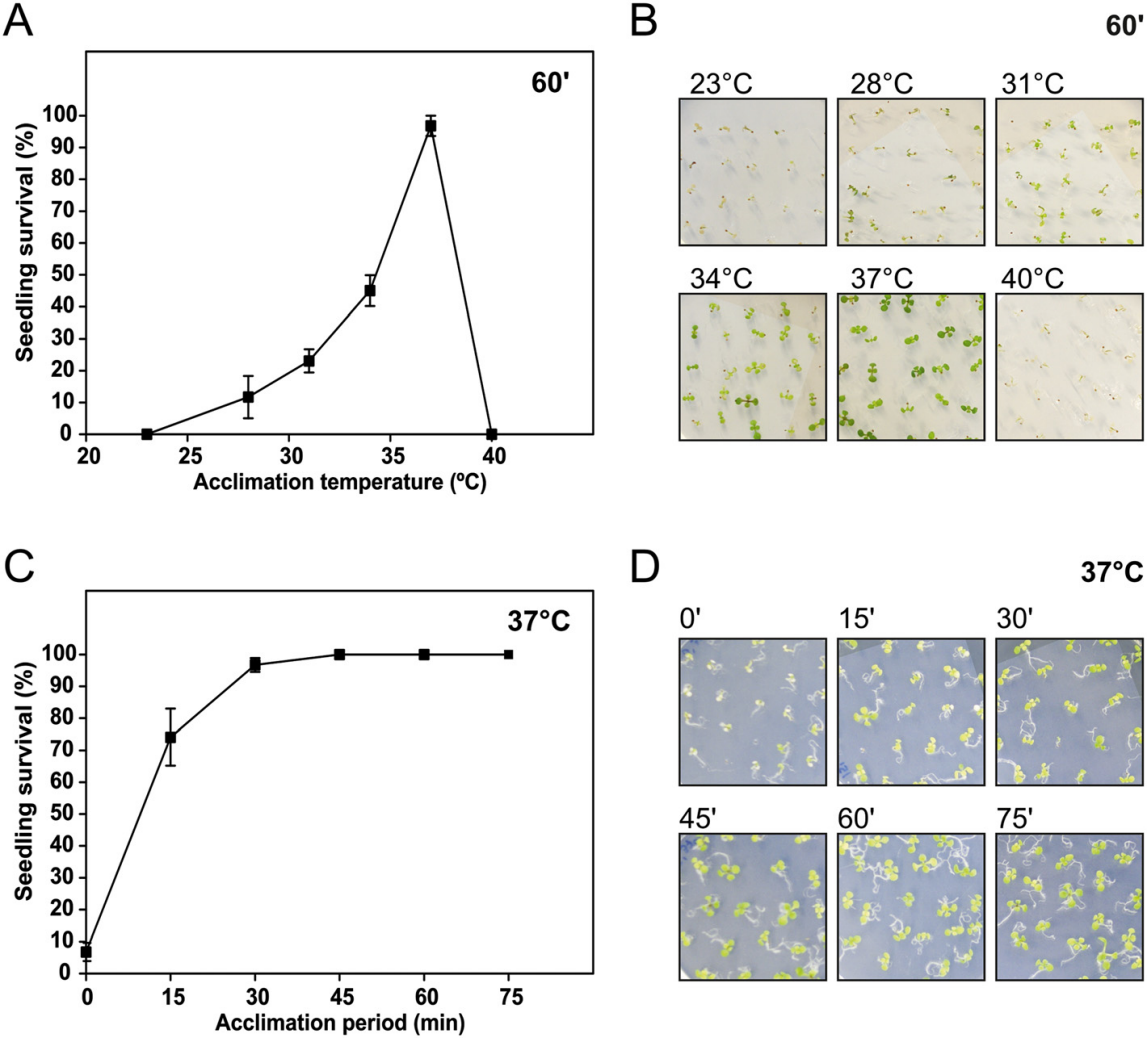
**Assessment of acquired heat tolerance in 7-day-old Col seedlings. (A-B)** Seedlings were acclimated for 60 min in a range of different temperatures (23-40°C), recovered at 23°C for 120 min and heat-treated at 45°C for 20 min. **(C-D)** Seedlings were acclimated at 37°C for different time periods (0–75 min), recovered at 23°C for 120 min and heat-treated at 45°C for 20 min. Seedling survival was scored after a 6-day recovery period, and represented as a percentage of initial viable seedlings. Error bars represent SEM (n = 5 plates with ~25 seedlings each).

To characterize acquired heat tolerance in Arabidopsis, 7-day-old Col seedlings were subjected to a range of acclimation temperatures (23-40°C) for 60 min, followed by recovery for 120 min at 23°C, prior to a 45°C and 20 min HS, which was previously established as an eminently lethal shock. Results indicate that acclimation efficiency positively correlated with acclimation temperatures up to 37°C, when survival was ~97% (Figure 2A,B). Subsequently, the acclimation capacity was quickly lost. We also performed a time-dependent analysis of a 37°C acclimation shock (Figure 2C,D). Results demonstrate that 30 min of acclimation resulted in viability of most seedlings, but a more extended period guaranteed complete survival of seedling to a subsequent heat shock. They also evidenced the sub-lethal nature of the 37°C treatment, since heat shock treatments for up to 75 min did not result in viability loss.

### Evaluation of basal heat tolerance through seed germination

We just described how basal and acquired heat stress responses could be analysed by seedling survival, modulating the intensity of the stimulus using ranges of temperatures and exposure times (Figures 1 and 2). However, this experimental design can be laborious when facing for instance different temperatures and an adequate number of replicas. To simplify the search for heat resistance and sensitivity phenotypes, we resorted to seed germination-based assays. As seeds normally exhibit higher heat tolerance than seedlings [19], the temperature and duration of treatments were altered accordingly.

**Figure 3:**
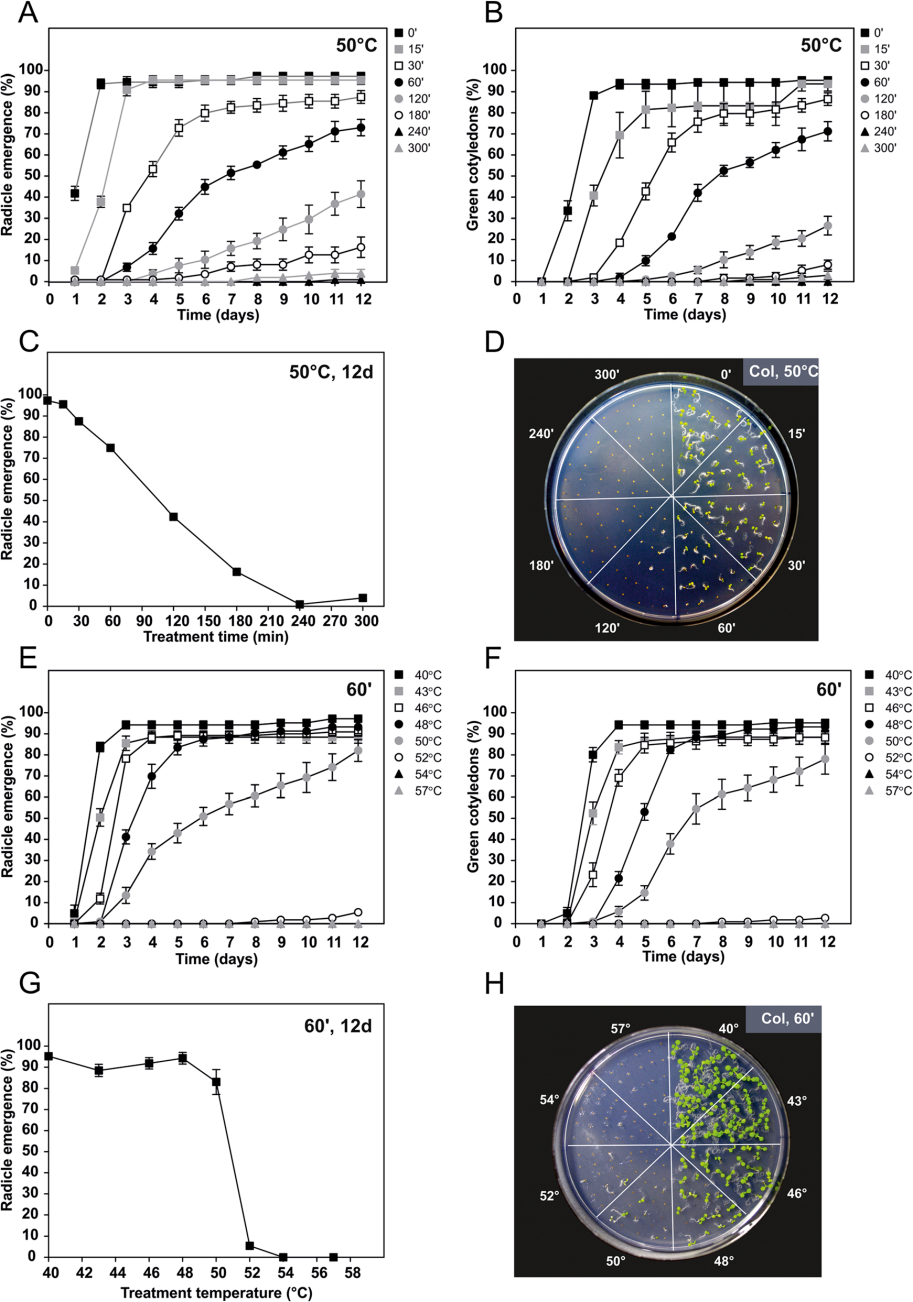
**Assessment of basal heat tolerance in Col seeds. (A-D)** Seeds were subjected to 50°C heat shock for different time periods (0–300 min). **(A)** Radicle emergence and **(B)** green cotyledon emergence during the 12 day post-heat shock period. **(C)** Germination levels (radicle emergence parameter) after 12 days, plotted as a function of treatment period. **(D)** Morphology of 12-day-old seedlings. **(E-H)** Seeds were subjected to a 60 min heat shock in a range of different temperatures (40-57°C). **(E)** Radicle emergence and **(F)** green cotyledon emergence during the 12 day post-heat shock period. **(G)** Germination levels (radicle emergence parameter) after 12 days, plotted as a function of heat-shock temperature. **(H)** Morphology of 12-day-old seedlings. Percentages were determined in relation to the total number of sown seeds. Error bars represent SEM (n = 5 plates with ~20 seedlings each).

A thorough map of the Arabidopsis seed germination response to heat shock imposition was once more performed in the wild-type ecotype Col. Seed heat tolerance assays were performed either with increasing periods (0–300 min) of heat shock treatment at 50°C (Figure 3A-D), or with increasing temperatures (40-57°C) of a 60 min treatment (Figure 3E-H). Results were highly consistent between radicle emergence and green cotyledon indicators, with an approximate 2-day delay between parameters. As expected, germination was compromised in both a time-dependant and temperature-dependent manner. By profiling germination on a daily-basis for up to 12 days, we observed that seeds tended to recover from HS with time rather than quickly display their full germination capacity (Figure 3A,B,E,F). We chose to calculate radicle emergence after 12 days, as a function of exposure time and temperature variables (Figure 3C,G). Results show that HS generated a sigmoidal-like inhibition curve for both time and temperature variables. HS duration immediately impacted on the germination capacity, generating a gentle time-dependent inhibition slope (Figure 3C). Meanwhile HS temperature generated a more dramatic inhibition, at ~50°C, with the germination rate remaining impervious to HS up until 48°C, yet diminishing almost 80% within a 2°C interval (between 50-52°C).

### Impact of natural variability and functional mutations on seed heat stress resistance

**Figure 4:**
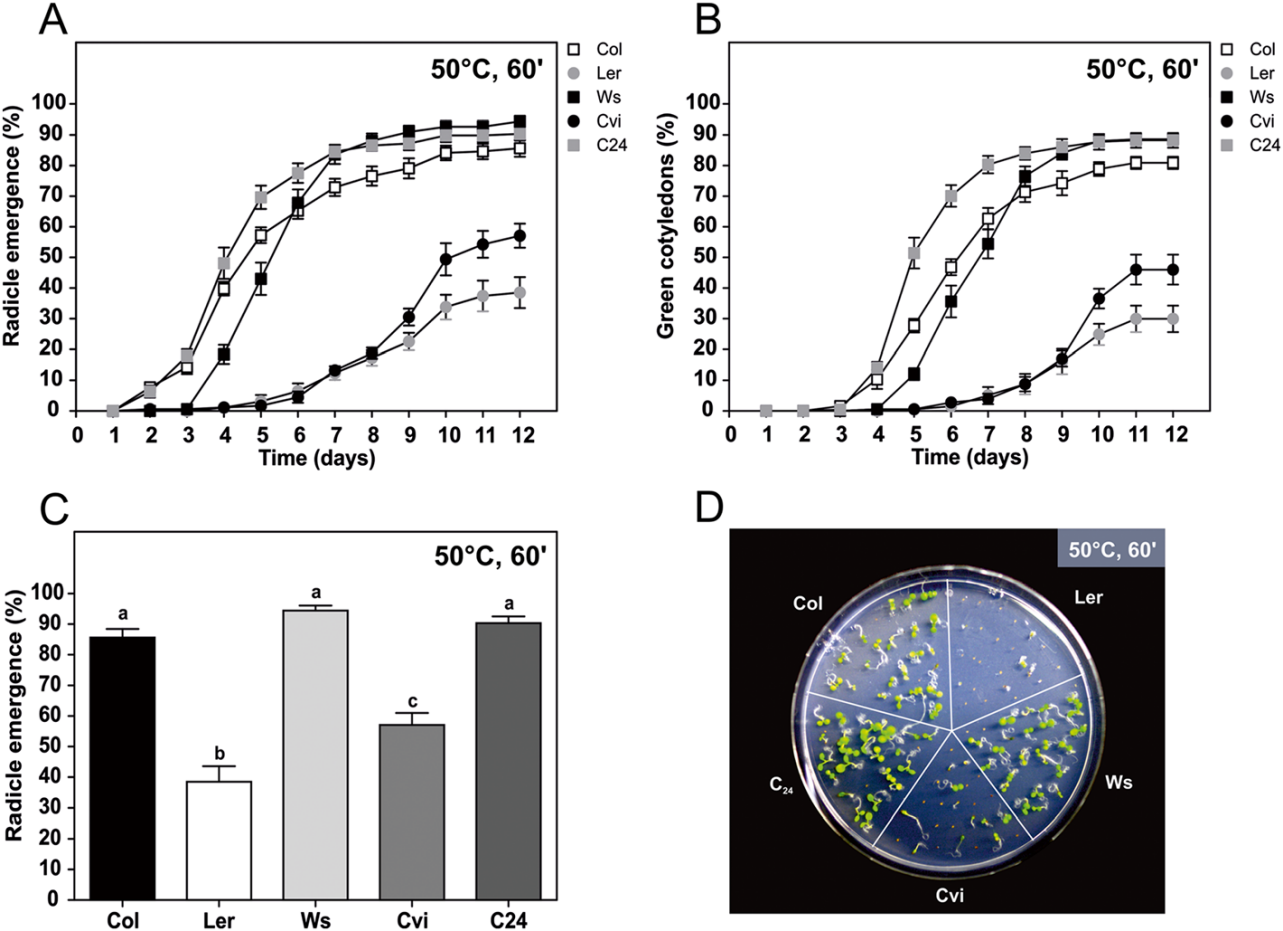
**Assessment of seed basal heat tolerance in different *Arabidopsis thaliana *ecotypes. (A-D)** Seeds were subjected to a 50°C heat shock for 60 min. **(A)** Radicle emergence and **(B)** green cotyledon emergence during the 12 days post-heat shock period. **(C)** Germination levels (radicle emergence parameter) after 12 days. **(D)** Morphology of 12-day-old seedlings. Percentages were determined in relation to the total number of sown seeds. Error bars represent SEM (n = 7 plates with ~25 seedlings each). In C, letters represent statistically different genotypes (One-way ANOVA with Tukey’s multiple comparison test for all pairs of columns; P < 0.05).

To validate the current approach, we tested the tolerance of seeds to HS (50°C and 60 min) in various different genetic backgrounds. First, five different ecotypes were analysed to determine the assay’s suitability to natural variability studies (Figure 4). As determined by radicle emergence and formation of green cotyledons after 12 days, ecotypes WS and C24 were more resistant to HS, with ~90% germination, immediately followed by Col (~85%). Ecotypes Cvi and particularly Ler were significantly more sensitive to heat stress. Meanwhile, analysis on a daily basis allowed for the establishment of informative profiles (Figure 4A,B). For instance, C24 displayed early germination in comparison to WS, suggesting it is the most heat resistant ecotype. However, statistical analysis of germination after 12 days placed Col, WS and C24 as identically tolerant ecotypes (Figure 4C). Conversely, the initial profile of Cvi and Ler suggested equal responses to HS, which were only resolved later in the assay and therefore deemed statistically different. As before, green cotyledon estimation was as informative as radicle emergence (Figure 4B). Interestingly, germination profiles using the green cotyledon indicator seemed to stabilize at the end of the experiment (days 10-12), indicating that a 12-day analysis is suitable to resolve germination phenotypes in what concerns the heat stress response.

**Figure 5:**
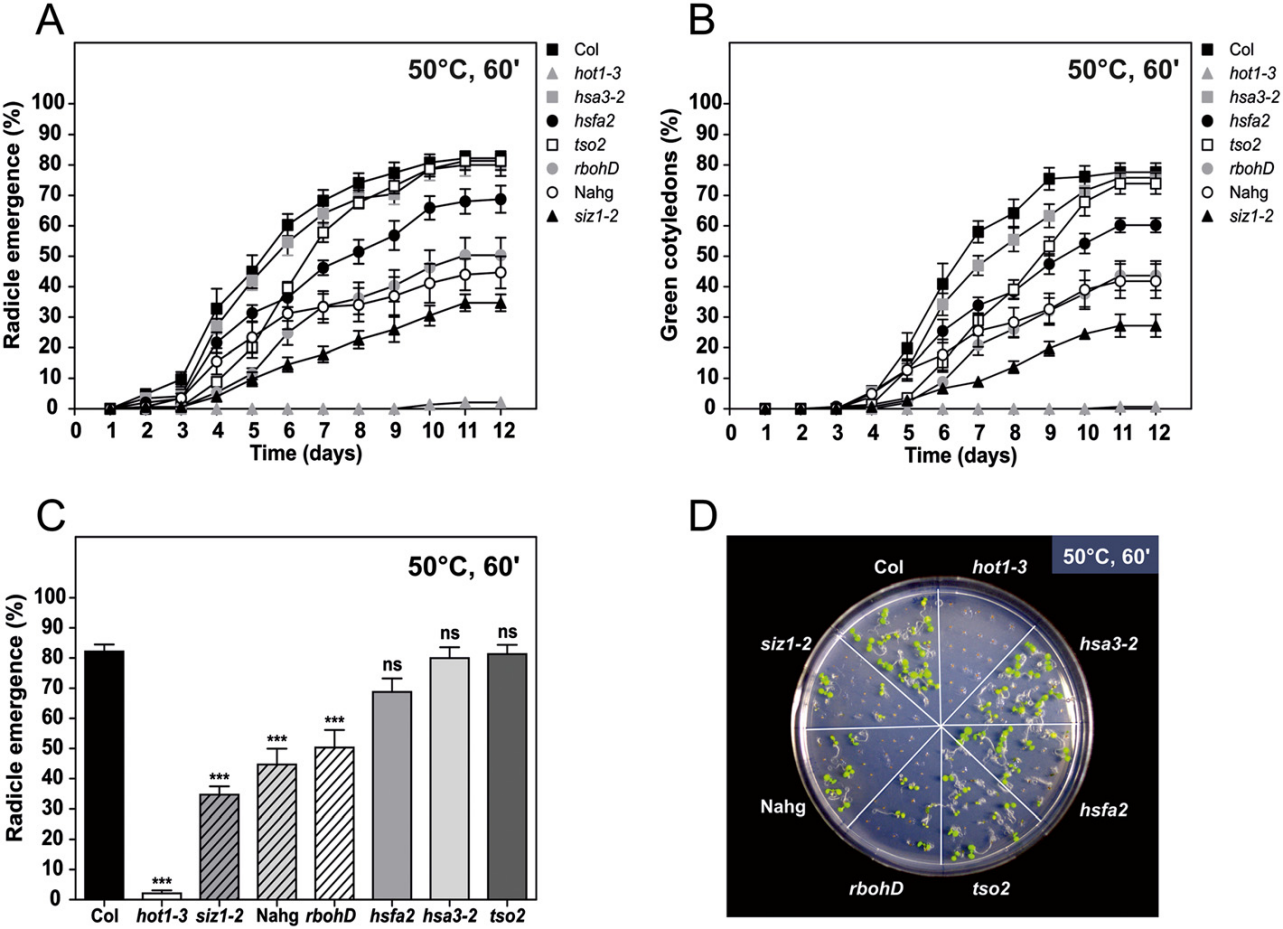
**Assessment of seed basal heat tolerance in different *Arabidopsis thaliana *functional mutant genotypes. (A-D)** Seeds were subjected to a 50°C heat shock for 60 min. **(A)** Radicle emergence and **(B)** green cotyledon emergence during the 12 day post-heat shock period. **(C)** Germination levels (radicle emergence parameter) after 12 days. **(D)** Morphology of 12-day-old seedlings. Percentages were determined in relation to the total number of sown seeds. Error bars represent SEM (n = 7 plates with ~25 seedlings each). Asterisks represent mutant genotypes with statistically significant differences in relation to the wild-type Col (Student *t*-test; ns, non-significant; ***, P < 0.001).

In parallel, we implemented the same strategy on functional mutants, consisting mostly of null alleles of known heat stress-related Arabidopsis genes. As a negative control, the mutant *tso2*, defective on the ribonucleotide reductase small subunit gene, was assayed in parallel. Mutant seeds subjected to a 50°C and 60 min HS were differentially affected by heat stress (Figure 5), and we were able to categorize mutants according to their sensitivity. Mutants were termed as highly sensitive (*hot1-3*), virtually losing seed germination capacity; moderately sensitive (*rbohD*; *NahG*; *siz1-2*), displaying 30-70% germination capacity; or insensitive to heat (*hsa3-2*; *hsfa2*), when germination rates within 12 days were statistically similar to the wild-type. As expected, the negative control mutant *tso2* displayed the same heat resistance level as the wild-type. As with previous results, germination rates stabilized within 12 days, validating this approach. However, daily scoring of the germination profile once again proved to be informative. For instance, the insensitive mutant *tso2* displayed a significant delay in germination though final germination rates were similar to the wild-type; the insensitive mutant *hsfa2* displayed a consistently slight defect in germination that did not overcome statistical analysis at day 12 (Figure 5A,B) but was statistically different when considering the time-course germination profile (paired Student *t*-test; data not shown).

## Discussion

Uniformity in plant trait measuring is an important aspect of current plant research [5], being equally valid for the analysis of developmental and stress-based traits. In the current report, several phenotypic assays were carried out to map changes in the heat tolerance capacity of Arabidopsis. The Col ecotype, which has become the ecotype standard within the Arabidopsis community and now congregates most functional studies [2], was characterized in what concerns seedling basal and acquired heat tolerance, as well as seed basal heat tolerance. These responses were analysed systematically, exploiting the two defining variables in a heat shock: temperature and exposure time. Later, seed-based analysis was validated using both Arabidopsis natural occurring ecotypes and functional mutants.

### Seedling-based assays for basal and acquired heat tolerance

A basic aspect to heat-related phenotype assays is the range of temperatures and treatment length. Studies in *Arabidopsis thaliana* normally specify 45°C as the standard temperature to detect alterations in plant fitness [6-9,11-13,17-19,25]; however, the duration of the heat treatment is highly variable in these reports, ranging from 15 to 220 min. Moreover, the reason why this temperature is the most commonly applied is not completely understood, since no extensive assessment of heat tolerance in Arabidopsis wild-type plants has been performed to the best of our knowledge. In the present report we showed that the 20 min heat shock period was the minimum necessary input to obtain nearly full lethality in Col. Lethality is likely the result of non-reversible denaturation of proteins or cellular structures [26,27]. A 45°C and 20 min treatment could thus be successfully used as a single shock treatment to resolve resistance in basal heat tolerance that didn’t overextend on the stress stimulus. The use of time- and/or temperature-dependant inhibition profiles, as performed in the present study, is even more suited to resolve basal heat resistance or hypersensitivity responses. Even though less exposure time may help resolve hypersensitivity to heat, the sensitivity of the method to slight variations in temperature or time, as depicted by the rapid inhibition curves, could compromise the establishment of a real heat phenotype.

A conditioning pre-treatment with moderate temperatures prior to a lethal temperature treatment has been shown to partially improve survival in several plant species [14,23]. This phenomenon, known as acquired heat tolerance, enables plants to withstand excessively high temperatures that would be damaging or lethal without pre-acclimation [26]. In the current work, variable preconditioning temperatures and exposure times were tested in Arabidopsis ecotype Col, using a subsequent recovery period of 120 min at 23°C and the predetermined experimental heat shock conditions (45°C for 20 min). By varying acclimation temperatures, we observed a very clear peak in seedling survival associated with pre-conditioning at 37°C. This would suggest that a significant heat stress input, in this case 15°C above the optimum growth temperature of Arabidopsis, is required to fully trigger signalling events leading to pre-acclimation responses. Also, tested pre-acclimation temperatures higher than 37°C (40°C) generated sufficient damage to compromise viability, confirming previous reports for the use of 37-38°C as the standard pre-acclimation temperature [7,14]. In support of the sub-lethal nature of a 37°C treatment, exposure of seedlings to this temperature over extended periods (higher than 45 min) did not induce any decrease in seedling viability.

As previously detailed, we demonstrated the critical necessity for a controlled experimental set up. Technically we suggest testing to be carried out by immersion of sealed plates in a water bath, as opposed to the use of heat chambers. We observed that heat transfer is significantly more effective over water in comparison to air (not shown). This procedure also avoids the loss in temperature that results from opening heat chambers, leading to underestimation of the stress input. In support, from three described heating devices (heating blocks, water baths, and growth chambers/ovens), Yeh and co-workers [21] also conveyed the water bath as the most efficient method to deliver heat stress temperatures. We additionally suggest that piling up plates be avoided, as it prevents adequate heat transfer, generating significant variability within replicates (not shown).

### Seed-based assays for basal heat tolerance

Seed-based assays provide an alternative approach to the analysis of heat stress resistance or hypersensitivity. They are less laborious and allow the analysis of a higher number of plants or genotypes. They also overcome most of the previously referred problems in delivering heat shock in a precisely controlled manner, by allowing treatments to be performed in microtubes. We highlight the importance of performing experiments using synchronized seed stocks to avoid variability in seed germination and fitness.

In seeds, heat challenges can affect both the overall germination capacity and germination progression. In the present report, when germinating seeds were daily scored, slower germination profiles were observed as a function of stronger treatments, suggesting a correlation between heat stress intensity and seed dormancy. As the germination process is defined as the set of events occurring after imbibition until a visible protrusion of the radicle occurs [28], a seed was considered germinated when emergence of the radicle was observable. Often however, formation of green cotyledon leaves is considered as the germination parameter [29]. Present results were consistent between both parameters with an observable 2-day delay for green cotyledon formation, suggesting that heat stress affects seed dormancy/germination but not photomorphogenesis of green cotyledons.

Even though plant trait phenotyping requires extremely controlled conditions [5], an often underestimated aspect is the time point at which plant traits are scored. Daily estimation of germination rates can facilitate phenotype discovery, but for a less laborious approach, we suggest data acquisition after 12 days, as the germination capacity became stabilized following that period for a significant number of genotypes and heat stress intensities. Consistent with previous reports [19], seed lethality required a significant increase in stress intensity compared to seedlings. For recognition of basal heat stress phenotypes, we propose evaluation of seed germination by varying shock time (50°C; 15–180 min) rather than shock temperature (47-53°C; 60 min), as it provides a gentler inhibition slope that facilitates phenotype discovery and mathematical establishment of EC_50_-like parameters. When facing experimental limitations, a 50°C and 60 min shock can be adopted as standard to amply resolve sensitivity to heat while maintaining sufficient resolution to identify heat stress resistance phenotypes.

### Addressing natural variability and functional heat stress-related mutant phenotypes

The ability to detect differential phenotypes using seed-based assays was validated using Arabidopsis ecotypes from various geographical locations. Differences can be associated to the physiological variation of individual ecotypes [30] with ecotypes Landsberg erecta (Ler) and Cape Verde Islands (Cvi) clearly manifesting higher sensitivity to heat stress. The observed variability can be attributed to the complex admixture patterns that have been associated to *Arabidopsis thaliana*, as a consequence of the recolonization of Europe following the last glaciation [31]. The enclosed methodologies were also designed to help screen for phenotype differences in mutants that have altered basal or acquired heat tolerance responses. In particular, seed-based phenotypic assays were sufficiently sensitive to characterize various degrees of basal heat tolerance, as mutants were easily resolved between insensitive, moderately sensitive or highly sensitive to heat stress imposition. Specifically, *hot1-3* was significantly affected by high temperature, confirming previous evidence that implicated *HOT1*, a major heat shock protein, in both basal and acquired heat tolerance [20]. Conversely, loss-of-function mutants for *HSFA2* (heat shock transcription factor) and *HSA32* (heat stress associated protein), both functionally associated to acquired but not to basal resistance [6,7], presented basal heat tolerance similar to the wild-type and the negative control mutant *tso2* (ribonucleotide reductase small subunit gene). Moderate sensitivity to heat stress was observed for *NahG*, a mutant ectopically expressing a bacterial salicylate hydrolase that scavenges salicylic acid [32]; the null allele of *SIZ1*, a major SUMO E3 ligase that is involved in several abiotic stress responses [33]; and *RBOHD*, the main NADPH oxidase in Arabidopsis, involved in ROS generation and signalling [34]. Results support previous plant level evidence for *NahG* and *rbohD* sensitivity to basal and acquired heat tolerance and *siz1-2* sensitivity to basal but not acquired heat tolerance ([9], reviewed by [33]). Interestingly, the present analysis was able to resolve a potential basal seed heat tolerance phenotype in *rbohD* which was undetected in a previous analysis by Larkindale et al. [9].

## Conclusions

The proper characterization of basal and acquired heat tolerance is crucial to address the molecular aspects underlying heat stress resistance. For instance, several QTLs related to germination have been reported in different crops (e.g. [35]) and in model plants such as Arabidopsis [36] and *Medicago truncatula*[37]. From these studies several candidate genes have been identified that could be associated with germination in high temperatures (e.g. [37] and references therein). These traditionally conform to the seven canonical classes of proteins associated with heat stress responses in eukaryotes [38]. The use of model plant species, such as *Arabidopsis thaliana*, will speed up the identification of more key determinant genes, either underlying QTLs related to germination, or involved in heat resistance mechanisms at plant level. For such studies the use of a simple set of protocols to detect seed and plantlet thermotolerance will be greatly beneficial. Also, the outlined methodologies proved to be effective in facilitating the detection of variability amongst geographically distinct natural accessions. In this context, they can be extended to studies addressing allelic variability in Arabidopsis, which are being potentiated by recent NGS-based efforts such as the *Arabidopsis thaliana* 1001 Genomes Project [31].

The present assays provide a framework of experimental conditions that are also helpful as an inexpensive and undemanding approach for functional studies. They are particularly appropriate for reverse genetic strategies that require sensitive phenotyping in the search for elusive phenotypic traits in the researcher’s mutants-of-interest. A comprehensive discussion of the diverse heat stress phenotyping methods and their importance for plant molecular genetic studies has already performed elsewhere [21]. Although the complete understanding of heat stress responses should consider different heat stress regimens and evaluation of different output traits [21], by focusing on the establishment of a set of heat response protocols we hope to provide a simple and standard way of detecting thermotolerance phenotypes.

The utility of plate-based analysis has been highlighted in the mapping of developmental phenotypes [5]. By using plate-based assays, one can detect early phenotypes that are often overlooked in soil-based phenotypic analyses, while simultaneously minimizing natural variability. Furthermore, time consumption and space requirements are maximized, making data collection amenable to high throughput experimental set-ups such as forward genetic screens. The basis for the isolation of novel loss- and gain-of-function mutations that are determinant for heat stress resistance is thus facilitated.

## Methods

### Plant material

Wild-type seeds of *Arabidopsis thaliana* ecotypes Columbia (Col), Wassilewskija (Ws), Landsberg erecta (Ler), Cape Verde Islands (Cvi) and C24 were ordered from the Nottingham Arabidopsis Stock Centre (NASC) (http://arabidopsis.info/; [39]). Homozygous T-DNA insertion mutant seeds of *hot1-3*[20] and *atrbohD*[40] were kindly provided by E. Vierling (Department of Biochemistry & Molecular Biophysics, University of Arizona, USA) and M. A. Torres (Department of Biology, University of North Carolina, USA), respectively. An RNAi line of *Hsa32*[7] and the *tso2* mutant [16] were kindly provided by Y. Charng (Agricultural Biotechnology Research Center, Academia Sinica, Taiwan) and J. Burke (USDA-ARS Cropping Systems Research Laboratory, USA), respectively. T-DNA insertion line of *HsfA2* (SALK_008978) was obtained from the Arabidopsis Biological Resource Center (ABRC; Ohio State University). The T-DNA insertion mutant *siz1-2* (SALK_065397) was ordered from the NASC European Arabidopsis Stock Centre (arabidopsis.info). The transgenic line *NahG*, that expresses a bacterial SA hydroxylase, was kindly provided by Miguel Botella (University of Malaga, Spain). All genotypes were grown in synchronized fashion and bulk seed stocks were maintained in the dark, at room temperature, in firmly sealed individual containers to prevent rehydration.

### Seed sterilization and in vitro germination

Arabidopsis seeds were stratified by immersion in water and incubation at 4°C for 3 days. Surface-sterilization was initiated by soaking seeds in 1 ml of 70% (v/v) ethanol for 5 min. Ethanol was replaced by 1 ml of bleach solution [20% (v/v) commercial bleach with 3.5% (w/v) effective chloride; 0.1% (v/v) Tween-20] and seeds were further incubated for 10 min. After being rinsed five times with sterile water, seeds were resuspended in a sterile 0.08% (w/v) agarose solution. When required, seeds were subjected to heat shock. Sterilized seeds were dispersed onto plates containing MS-based medium [41] composed of 1x basal salt mixture (Duchefa), 1.5% (w/v) sucrose, 0.5 g l^−1^ MES and 0.8% (w/v) agar at pH 5.7. Plates were sealed with parafilm to prevent dehydration and placed horizontally in a growth room, under the following conditions: photoperiod of 16 h light/8 h darkness; light intensity of 100 μmol photon m^−2^ s^−1^ and 23°C.

### Seedling survival assays

Seeds were plated onto MS medium and grown horizontally under the same conditions as previously referred. After 7 days of growth, heat stress was imposed by submersion of parafilm-sealed plates into a temperature-controlled water bath. Heat treatment was performed 3 h into the light/day photoperiod, under a dim light. Four kinds of assays were performed: a) treatment at different extents of time (5–45 min) under a constant temperature of 45°C; b) incubation at different temperatures (38-50°C) for 20 min; c) pre-incubation at different temperatures (28-40°C) for 60 min, recovery at 23°C for 120 min, and heat-treatment at 45°C for 20 min; d) pre-incubation at different extents of time (15–75 min) under a constant temperature of 37°C, recovery at 23°C for 120 min, and heat-treatment at 45°C for 20 min. After heat treatments, seedlings were returned to the growth room and allowed to recover for 6 days. The number of viable seedlings was then quantified to determine the survival rate. Seedlings that were still green and generated new leaves were scored as survivors.

### Germination assays

Seed heat tolerance assays were performed immediately after stratification and sterilization. Seeds were heat-stressed by submersion of the corresponding microtubes into a temperature-controlled water bath. Three sets of assays were performed: a) treatment of Col at different extents of time (0–300 min) under a constant temperature of 50°C; b) incubation of Col at different temperatures (40-57°C) for 60 min; c) treatment of different ecotypes and functional mutants at 50°C for 60 min. Subsequently, seeds were plated onto MS medium and monitored daily for germination (radicle emergence and formation of green cotyledons) over a 12-day period. The germination frequency was determined in relation to total sown seeds per each condition.

## Abbreviations

HS: Heat shock; ROS: Reactive oxygen species; NGS: Next-generation sequencing.

## Competing interests

The authors declare that they have no competing interests.

## Authors’ contributions

JSC and SF carried out all the experiments and drafted the manuscript, RMT participated in the study design and contributed to data analysis, TLN conceived the study, contributed to its design and revised critically the manuscript, HA coordinated the study, contributed to the design, analysis and interpretation of data and revised critically the manuscript. All authors read and approved the final manuscript.
